# Influence of pupillary dynamics on the defocus curve of eyes implanted with diffractive multifocal lenses: a randomized study

**DOI:** 10.5935/0004-2749.20210029

**Published:** 2021

**Authors:** André Messias, Miriam Ferreira, Gleilton Carlos Mendonça, Wilian Queiroz, Roberto Pinto Coelho, Katrin Gekeler

**Affiliations:** 1 Departamento de Oftalmologia, Otorrinolaringologia e Cirurgia da Cabeça e Pescoço, Faculdade de Medicina de Ribeirão Preto, Universidade de São Paulo, Ribeirão Preto, SP, Brazil; 2 Department of Ophthalmology, Klinikum Stuttgart, Stuttgart, Germany

**Keywords:** Multifocal intraocular lenses, Pupil/physiology, Cataract, Phacoemulsification, Lentes intraoculares multifocais, Pupila/fisiologia, Catarata, Facoemulsificacão

## Abstract

**Purpose:**

To evaluate the influence of pupil dynamics on the defocus profile and area-of-focus of eyes implanted with a diffractive multifocal intraocular lens (IOL).

**Methods:**

This prospective randomized trial was conducted at the Department of Ophthalmology, School of Medicine of Ribeirão Preto, University of São Paulo, Brazil. Thirty-eight patients were randomly assigned to receive the multifocal SN6AD1 (n=20) or the aspheric monofocal SN60WF (aIOL) (n=18) IOLs bilaterally. Dynamic pupillometry, visual acuity for distance and near, corrected and uncorrected, and a defocus profile were assessed postoperatively. The area-of-focus was calculated using an empirical polynomial model of the defocus profile.

**Results:**

Sixteen patients (32 eyes) in the multifocal SN6AD1 group and 17 patients (34 eyes) in the aspheric monofocal SN60WF group completed the 1-year follow-up. There were no significant between-group differences in monocular uncorrected distance or near visual acuity. The defocus profiles of the mfIOL group showed a double peak, whereas those of the aspheric monofocal SN60WF group showed only one peak, which is typical for a monofocal intraocular lens. The area-of-focus of the aIOL group (4.66 ± 1.51 logMARxD) was significantly different from that of the multifocal SN6AD1 (1.99 ± 1.31 logMARxD). Pupil size at maximum contraction after exposure to a flash of 30 cd/m^2^ for 1 second was significantly correlated with a better area-of-focus in the multifocal SN6AD1 group (r=0.54; p=0.0017), whereas this was not the case in the aspheric monofocal SN60WF group.

**Conclusion:**

These findings indicate that in eyes implanted with an multifocal SN6AD1, the smaller the pupil size, the better is the area-of-focus and hence the better is the visual performance. This correlation was not found for the aspheric monofocal SN60WF.

## Abstract (pt)

**Objetivo:**

Avaliar a influência da dinâmica pupilar na curva de desfoco de olhos implantados com lente intraoculares multifocais difrativas.

**Métodos:**

Estudo prospectivo e randomizado realizado na Faculdade de Medicina de Ribeirão Preto - Universidade de São Paulo - Departamento de Oftalmologia. Trinta e oito pacientes foram aleatoriamente designados para receber bilateralmente lentes intraoculares SN6AD1 (n=20) (mfIOL) ou SN60WF (n=18) (aIOL). Além da acuidade visual para longe e perto, corrigida e não corrigida, e curva de desfoco, foi ainda realizada pupilometria dinâmica. A área sob a curva de desfoco foi calculada usando um modelo polinomial empírico.

**Resultados:**

Um total de 16 e 17 pacientes (n=32 e 34 olhos) completaram 1 ano de seguimento nos grupos mfIOL e aIOL, respectivamente. Não houve diferenças significativas entre grupos para as acuidades visuais seja para longe ou perto. As curvas de desfoco do grupo mfIOL mostraram um pico duplo; enquanto o SN60WF mostrou apenas um pico, típico para uma lente intraoculares monofocal. A média da área sob a curva de desfoco do grupo aIOL foi (4,66 ± 1,51 logMAR.dp), e essa é estatisticamente significante diferente da métrica do grupo mfIOL (1,99 ± 1,31 logMAR.dp). A pupila na contração máxima após a exposição a um flash de 30 cd/m^2^ por 1 segundo foi significativamente correlacionada com uma melhor área de foco no grupo mfIOL (r=0,54; p=0,0017), essa relação não foi observada para o grupo aIOL.

**Conclusão:**

Estes dados indicam que quanto menor a pupila durante contração, melhor é a área sob a curva de desfoco e, portanto, o desempenho visual dos olhos implantados com essa mfIOL. Esta correlação não foi encontrada para lentes intraoculares monofocais.

## INTRODUCTION

Modern diffractive multifocal intraocular lenses (mfIOLs) are widely used to restore far and near visual function after cataract extraction, thus providing independence of glasses, with several scientific reports showing promising outcomes^(1-5)^. However, the implantation of mfIOL has been occasionally associated with patient complaints, particularly of low contrast sensitivity, intraocular straylight, and poor near and far visual acuity^(6-8)^.

Whereas pupil size with refractive mfIOLs may have an impact on visual acuity at different distances, this should not be the case for diffractive mfIOLs. On the other hand, large pupil sizes with diffractive multifocal intraocular lenses (IOLs) may have an impact on disturbing photic phenomena^(6)^.

A previous report correlated larger pupils with better distance visual acuity^(9)^, regarding the pupil as an aperture in the optical pathway. However, visual acuity at different distances may not represent the full range of vision that is possible with multifocal IOLs. The defocus profile seems to be a more precise way to analyze visual performance of different IOLs. On the other hand, quantification and measurement of the defocus profile may be a complicated task. Buckhurst et al. recently proposed an “area-of-focus” metric, a calculation based on the area of visual acuity within the range of the defocus profile, which provides a simple method of evaluating IOL defocus profiles^(10)^. In this study, the influence of a monofocal IOL, a diffractive mfIOL a refractive mfIOL, and a mix-and-match combination of two mfIOLs on the area-of-focus was described, while the influence of pupil size on the area-of-focus metric of mfIOL was not evaluated^(10)^. In this context, we aimed to evaluate the influence of pupil size under photopic conditions on the area-of-focus from eyes implanted with a monofocal aspheric IOL and an apodized diffractive multifocal IOL.

## METHODS

This interventional, prospective, randomized study was performed at the Department of Ophthalmology, School of Medicine of Ribeirão Preto, University of São Paulo, Brazil in 2012 and 2013.

The study protocol was approved by the Hospital Ethics Committee under process number 11843/2010, but the study was not registered as a clinical trial. The tenets of the Declaration of Helsinki were followed. Patients from the ophthalmology outpatient clinic at the Department of Ophthalmology who opted for bilateral phacoemulsification were screened to be eligible for the study. Informed consent was obtained from all patients. The exclusion criteria were any ocular disease other than cataract, corneal astigmatism >1 diopter (D), and spherical equivalent < - 3 D or > + 5 D. Patients were eligible for randomization if they had bilateral cataract and corneal astigmatism <1 D. Since these patients had no ocular disease except cataract, we expected similar postoperative far visual acuity in all groups. Therefore, the area-of-focus is reported as the primary outcome. This is a calculation derived from the defocus curves, and its variability was estimated retrospectively from patients implanted with SN6AD1 showing a mean of 3.0 logMARxD and a standard deviation of 1.4 logMARxD. Consequently, to achieve 80% power in detecting a between-group difference of 3 logMARxD, the calculated sample size would be 11 patients per group.

Preoperative examinations included a comprehensive ophthalmologic evaluation; optical coherence tomography (Spectralis, Heidelberg Engineering, Heidel berg, Germany); pupillometry, biometry, and IOL calculation (LensStar, Haag-Streit International, Köniz, Switzerland); and monocular and binocular, uncorrected and best corrected, far and near visual acuity. For visual acuity at distance, the Early Treatment of Diabetic Retinopathy Study (ETDRS) charts were used at 4 m. For near visual acuity, ETDRS modified Snellen charts (Lighthouse, Precision vision, Woodstock, Illinois, USA) at 30 cm were used.

The study comprised two different IOLs: the diffractive multifocal IOL SN6AD1 (mfIOL) and the aspheric monofocal IOL SN60WF (aIOL). Both are single-piece lenses with ultraviolet and blue light filtering capabilities. The SN6AD1 has an apodized diffractive pattern that results in an addition of + 3.0 diopters on the IOL plane. The anterior surface is designed with negative spherical aberration (-0.1 µm). The SN60WF is a monofocal IOL with an aspheric design (-0.2 µm) to compensate for the corneal spherical aberration.

The patients were randomly assigned to receive either a ReSTOR SN6AD1 (Alcon, Novartis, Freiburg, Switzerland) (n=20) or an SN60WF (n=20) bilaterally and were evaluated at baseline, postoperative days 1 and 10, and 1, 3, and 12 months after surgery. Phacoemulsification was performed under topical anesthesia through a 2.75-mm incision with a capsulorhexis of 5 to 5.5 mm. All IOLs were implanted in the capsular bag.

### Defocus

To determine the area-of-focus, defocus profiles (visual acuity over imposed defocus) were assessed by measuring monocular visual acuity at 4 m starting from distance correction. Visual acuity at 4 m was then assessed with added lenses in half-diopter steps from -5.00 to +3.00 D. Thus, 17 visual acuity measurements were performed for each eye in each patient to evaluate the defocus profile.

**Figure 2:**
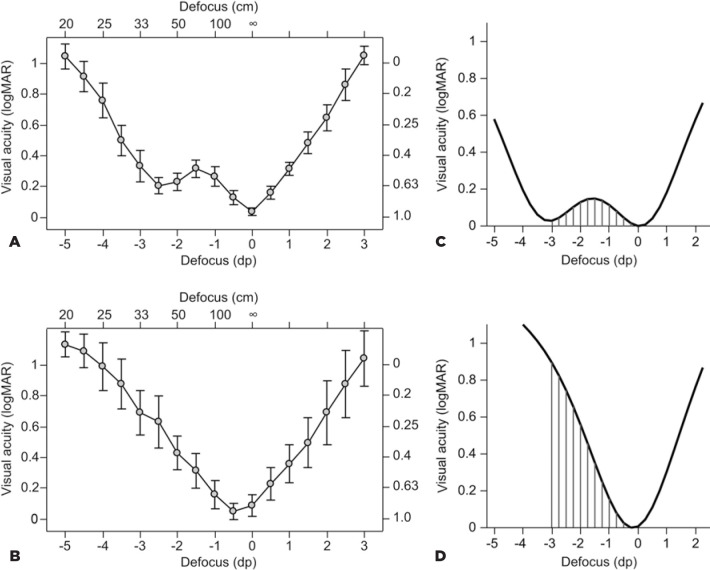
Mean defocus profiles of the mfIOL group (A) and the aIOL group (B). Examples of the empirical model for the mfIOL group (C) and the aIOL group (D) that were used to fit the defocus results as previously described^(2)^ and the calculation of the area-of-focus^(10)^. The calculated area-of-focus is the area under the defocus profile between the best far visual acuity and defocus of - 3 D.

An empirical model was used to fit the defocus results, as previously described^(2)^. Modeling the defocus profiles allowed calculation of the area under the curve as a metric that includes the assessment of far, intermediate, and near vision values (see Figure 2C and 2D for examples). In other words, the calculated area-of-focus is basically the sum of the visual acuities or the area under the defocus profile between the best far visual acuity and defocus of -3 D, and the higher the result, the worse is the defocus profile.

### Pupillometry

Pupillometry was performed after a short period of dark adaptation (at least 2 minutes) using a camera-based, commercially available, pupilometer (ISCAN, Woburn, MA, USA) with a 60-Hz frame rate. Light stimuli were generated using a ganzfeld light source (ColorDome, coupled to the control unit, the Espion E2- Diagnosys-LLC, Lowell, Massachusetts, USA), and consisted of 1-second flashes of 30 cd/m^2^. The pupilometer and the light source were synchronized by connecting the pupilometer output trigger to the Espion external input channel. The stimuli were repeated six times for averaging and rejection of potential blink artifacts, with a 5-second interstimulus interval. The sessions took approximately 10 minutes.

**Figure 1:**
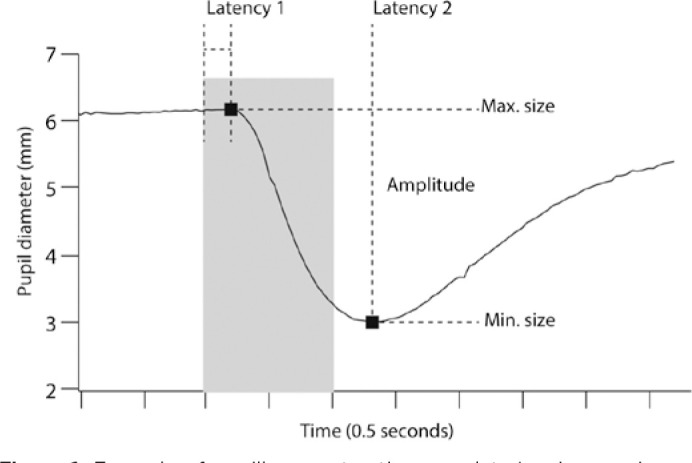
Example of pupillary contraction wavelet showing maximum and minimum pupil size, time between light stimulus on and beginning of contraction (latency 1), and time to maximum contraction (latency 2).

Each pupillary contraction wavelet was analyzed offline to reject blink artifacts and determine the following parameters: maximum and minimum pupil size, time between light stimulus on and beginning of contraction (latency 1), and time to maximum contraction (latency 2) (Figure 1).

Statistical analyses were performed using JMP IN software version 13.1.0 (SAS Institute, Cary North Carolina, USA). One-way analysis of variance (ANOVA) was used for group comparison of continuous variables. A *P*-value less than 0.05 was considered to indicate statistical significance. The 12-month results were used as postoperative values.

## RESULTS

Sixteen patients in the mfIOL group and 17 patients in the aIOL group completed the 48-week follow-up. Two patients in group aIOL decided not to complete the last two visits and were therefore excluded from the analysis.

As expected, due to the randomized protocol, there was no difference between the groups in preoperative corrected distance visual acuity (CDVA): 0.26 ± 0.02 logMAR for the mfIOL group and 0.30 ± 0.03 logMAR for the aIOL group (*P*=0.2893, ANOVA). There were also no significant differences between the groups in preoperative uncorrected distance visual acuity (UDVA) correction; or near, with or without correction (CNVA and UNVA), preoperative spherical equivalent or astigmatism (p>0.05).

Postoperatively, there was no significant difference between the groups in CDVA measured 1 month after the procedure (p=0.8724, ANOVA). The mean CDVA was 0.05 ± 0.02 and 0.03 ± 0.02 logMar for the mfIOL and aIOL groups, respectively. CDVA remained at these levels during 48 weeks of follow-up.

As a result of good predictive ability of the IOL calculation formulas, there was no significant difference between the groups in postoperative spherical equivalent, with -0.22 ± 0.40 D in the mfIOL group and - 0.29 ± 0.29 D in the aIOL group. The mean postoperative astigmatism at week 48 was - 0.66 ± 0.52 and - 0.71 ± 0.53 D in the mfIOL and aIOL groups, respectively. The mean monocular UDVA was 0.18 ± 0.02 and 0.14 ± 0.03 in the mfIOL and aIOL groups, respectively. The mean monocular UNVA was 0.08 ± 0.01 and 0.51 ± 0.05 in the mfIOL and aIOL groups, respectively; the value was significantly better in the mfIOL group (p=0.0031).

### Defocus

The defocus profiles of the mfIOL group showed the typical “double peak” of a multifocal (essentially bifocal) IOL (Figure 2A and 2C). The defocus profiles of the aIOL group showed only one peak (Figure 2B and 2D), which is typical for a monofocal IOL.

The mean area-of-focus was 1.99 ± 1.31 logMARxD in the mfIOL group and 4.66 ± 1.51 logMARxD in the aIOL group; the difference was statistically significant (p<0.001).

### Pupillometry

There was no statistically significant difference between the mean minimum pupil sizes of the mfIOL and the aIOL groups: 3.1 ± 0.1 and 3.2 ± 0.2 mm, respectively (p=0.7272). There were also no statistically significant differences between the groups in maximum and minimum pupil size, latency 1, or latency 2.

**Figure 3:**
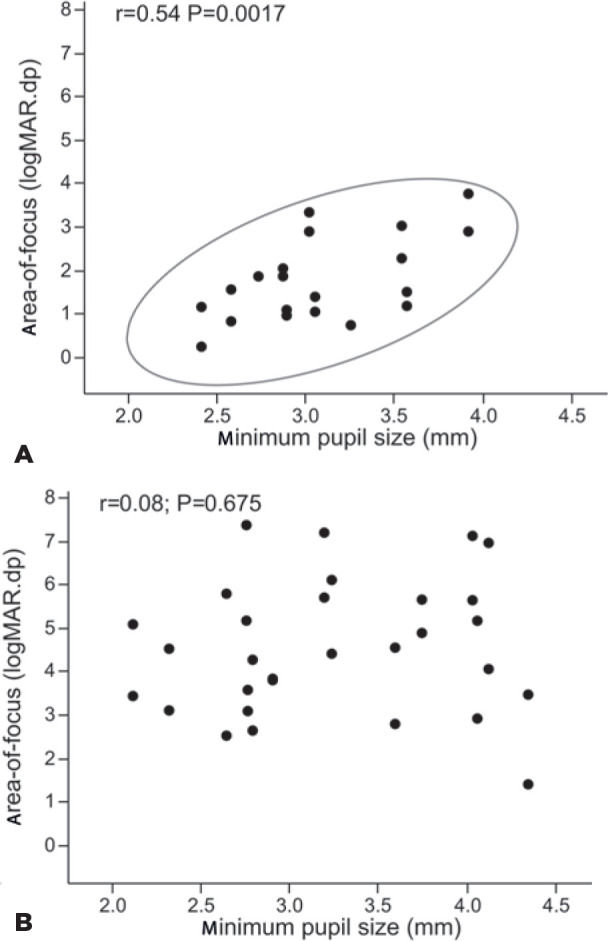
Correlation of the area-of-focus with minimum pupil size in the mfIOL group (*r*=0.54; *P*=0.0017) (A) and the aIOL group (*r*=0.08; *P*=0.675) (B).

Interestingly, the area-of-focus of the mfIOL group was significantly correlated with the minimum pupil size (r=0.54; p=0.0017) (Figure 3A). That is, the smaller the pupil size, the better the area-of-focus, and consequently the better the defocus profile. There was no significant correlation between minimum pupil size and area-of-focus in the aIOL group (r=0.08; p=0.675) (Figure 3B). This correlation of minimum pupil size and area-of-focus was not observed for the other pupillary parameters. Also, no correlations were found between pupillary contraction parameters and far or near visual acuity.

## DISCUSSION

Theoretically, optical quality measured by modulation transfer function and halo size, even of diffractive multifocal IOL, decreases as pupil size increases^(11)^. However, Santhiago et al. found no effect of pupil sizes of 6 or 4 mm on the modulation transfer function^(12)^. Thus, the effect of pupil size on optical quality after implantation of an mfIOL is controversial. These studies regard the pupil as an aperture in the optic pathway and not as a result of a complex neural pathway. On the other hand, clinical studies show different results. For example, Alfonso et al. reported better distance visual acuity and worse near visual acuity in patients with large pupils^(9)^. These results seem to be in contrast to our results, but the results are not comparable. Alfonso et al. reported statistically significant correlations between pupil size and visual acuity, with r=0.297 for distance visual acuity and *r*=0.276 for near visual acuity, which are not strong correlations^(9)^. In their study, pupil size was measured under illumination of 85 cd/m^2^, whereas in our study we measured the minimum pupil size at 30 cd/m^2^.

As Buckhurst et al. stated, comparisons of studies with multifocal IOLs are difficult^(10)^. Although measurements of far visual acuity may be standardized, measurements of near or intermediate visual acuity are not standardized at all. For example, the use of different reading charts and different distances makes the results of different studies not comparable.

The defocus profile should be taken as a standardized measurement, especially if visual improvement after mfIOL implantation should be evaluated. Furthermore, we believe that the described area-of-focus is an objective index of visual performance in eyes with such IOLs. We found that the smaller the pupil became after eyes were exposed to a strong light (30 cd/m^2^), the better was the area-of-focus in eyes implanted with an mfIOL.

Reasonably, under visual acuity testing or reading conditions, the pupil would be larger than during this experimental setting, so that pupil size might not be regarded as an aperture. Nevertheless, the fact that the pupil is able to contract to smaller sizes under strong light exposure indicates the health of a complex neuro-muscular system. For instance, individual pupil size is influenced not only by retinal or neural disorders but also by fatigue^(13)^, intelligence^(14)^, emotional state, or even music^(15)^. Training also has an impact on visual performance with mfIOLs^(16)^, while psychological characteristics have an impact on the perception of halos^(17)^.

Accordingly, we propose that studies regarding the pupil size of the patient as a simple aperture should simulate a pupil size with pinholes of different sizes^(18)^ rather than dividing patients into groups with different pupil sizes.

This study has a potential methodological limitation that is related to the measurement of defocus, which could also be measured binocularly, and to the small number of patients included. The study only shows the results for one special mfIOL, and therefore the results might not be extrapolated to other types of mfIOLs. More studies using the area-of-focus and pupillary dynamics assessment are needed to answer the question whether the showed correlation of minimum pupil size and area-of-focus für one special mfIOL is also the true for other IOLs.

In summary, we were able to show that the smaller the minimum pupil size under light exposure, the better the area-of-focus in patients implanted with an mfIOL (SN6AD1), but not in patients implanted with an aIOL (SN60WF).

### What was known

Pupil size may influence visual performance with multifocal IOLs. Until now, studies have regarded the pupil as a pinhole and analyzed visual acuity at different distances. A complete analysis of the defocus profile with an area-of-focus metric and correlation with pupil dynamics has not been performed.

### What this paper adds

The area-of-focus metric is a single value that quantifies the range of vision. This is the first study that correlates this value with pupil dynamics. The smaller the pupil becomes, the better the value of the area-of-focus.
